# New Concepts in Musculoskeletal Medicine

**DOI:** 10.3390/jpm14121162

**Published:** 2024-12-20

**Authors:** Patrick Haubruck

**Affiliations:** Raymond Purves Bone and Joint Research Laboratory, Kolling Institute, Institute of Bone and Joint Research, Faculty of Medicine and Health, The University of Sydney, St. Leonards, NSW 2068, Australia; patrick.haubruck@sydney.edu.au

The clinical relevance of musculoskeletal disorders (MSDs) continues to rise due to an ageing population and changes in lifestyle, and consequently this wide range of diseases and injuries remains a leading contributor to disability on a global scale [1]. Despite ongoing research and the resulting advances in the care of affected patients, challenges remain, creating an unmet medical need across the field of MSDs for a focused refinement of individual imaging modalities and surgical techniques. To achieve this refinement, interdisciplinary teamwork involving surgeons, researchers, biologists, engineers and computational scientists has become a “sine qua non”. Due to the increasing understanding of the fundamentals in the biology and pathology associated with MSDs, both researchers and clinicians are required to gain more and more specialized knowledge to address the unmet needs in their respective fields. Although honing this highly specialized knowledge has led to groundbreaking advances in patient care and paradigm-shifting research, keeping up to date with new findings in the wider field of MSD research remains crucial for all clinicians and researchers. This Special Issues serves as an ideal space to combine researchers from different areas of one medical field and to publish state-of-the-art findings. It serves as a great example by capturing the diversity in the field of MSDs, as it contains research investigating novel concepts in trauma- and reconstructive surgery, orthopaedic surgery, spinal surgery and MSD imaging techniques.

Large bone defects remain a challenge in modern reconstructive surgery, despite the introduction of the “Diamond concept”, which provides a framework of factors necessary to achieve bone union [2]. Based on this framework, the gold standard to achieve bone regeneration continues to be the use of an autologous bone graft, which in itself is a limited resource, and the process is associated with considerable morbidity. Thus, novel biomaterials, such as bioactive glass, have been developed and are currently being investigated as an expander of the autologous bone graft [3]. Findeisen et al. investigated the clinical efficacy of the combined use of autologous bone graft and bioactive glass (S53P4) for the treatment of large-defect-size non-unions of long bones of the lower limb (Contribution 1). Despite the challenging nature of the treated non-unions, the investigated treatment had a success rate of 69.2% after 2 years. When comparing these results with the literature, treatments such as bone transport might lead to a higher union rate [4]. However, as stated by Findeisen et al., the surgical revision rate in these treatments is high and thus can lead to a lower patient satisfaction. An important message from the authors is that revision surgery should not be performed as long as ongoing remodelling is detectable, as with each additional revision surgery the consolidation rate decreases [5].

Occult or chronic infections are known to inhibit bone regeneration, as well as causing a loosening of orthopaedic implants. Despite state-of-the-art infrastructure in modern operating rooms and considerable efforts to maintain a sterile environment, periprosthetic joint infection occurs in up to 1–2% of primary arthroplasties [6] and in approximately 5% after internal fixations of the bone fractures. While the detection of fulminant and acute infections can be performed based on clinical parameters, detecting occult infections is an ongoing challenge in the field of MSDs. The gold standard remains the sonication of the explanted implants and culturing of the fluid; however, this technology is not readily available in all hospitals and thus a technically less demanding alternative is needed. Here, Bakalakos et al. investigated the use of dithiothreitol (DTT), a soluble sulfhydryl compound, that can alter the extracellular matrix of a biofilm in order to free bacteria without requiring any further equipment and subsequently allow their detection via culturing [7]. The authors retrieved implants from a large cohort and divided them into two equal-length segments. Subsequently, they compared the accuracy of using DTT in comparison to the gold standard sonication in detecting occult infections. The results of this study showed a total concordance of 95.65% (Contribution 2), and therefore demonstrated non-inferiority, making DTT an alternative option, obviating the need for specialised equipment.

Minimizing surgical site infections as well as other potential complications is part of an ongoing refinement of surgical techniques. Surgical drains were developed to decrease post-operative hematoma and seroma that can act as local incubators for surgical site infections; however, in orthopaedic surgery there has been no consensus yet regarding the efficacy of surgical drains to reduce complications [8]. Adolescent idiopathic scoliosis (AIS) typically affects healthy individuals, and surgical treatment in progressive spinal curvature serves mostly an aesthetic purpose. Therefore, surgical treatment should be optimized to minimize the risks as much as possible to allow a favourable risk–benefit analysis of this treatment (Contribution 3). Consequently, Ruffilli et al. conducted a systematic review and meta-analysis of the literature investigating the effect of drains on short-term outcomes of AIS surgery. Included articles had a combined patient cohort of 772 patients, and detailed analysis of these articles revealed that no significant differences in short-term outcomes of AIS surgery were observed regardless of the usage of a drain. Thus, the choice of drainage continues to rely on the experience of the surgeon responsible.

This is an excellent example illustrating that despite having robust evidence, a variety of key decisions still need to be determined based on the experience of the surgeon. Therefore, ongoing research should seek to facilitate this intraoperative decision-making process by developing and establishing supporting tools. Primary total hip arthroplasty is well established as one of the most successful surgical treatments to date; however, leg length equalization is crucial and remains one of the most challenging targets (Contribution 4). Girolami et al. evaluated a simple compass device that is used with two anatomical landmarks to assess leg length intraoperatively. A total of 35 patients were included and leg length discrepancy (LLD) analysed pre- and post-operatively. Not only was a reduction in the postoperative LLD observed, but the average post-operative LLD was 2.5 mm, and therefore almost beneath the threshold of patient perception [9]; consequently, all patients were satisfied. Therefore, this study shows that a simple tool, which is both cost-effective and efficient, can still have a significant impact on the surgical treatment and overall patient outcome and satisfaction.

Finally, a further key aspect in refining surgical treatments and their associated outcome was identified as accurate pre- and postoperative imaging, enabling the surgeon to accurately correlate patient satisfaction with the surgical outcome. Revision total hip arthroplasties in the presence of severe acetabular bone defects remains a challenge in the field of MSD. Novel custom-made 3D implants provide a helpful solution; however, precise placement is critical for surgical success. Nees et al. compared conventional X-rays with more advanced 3D CT scans for assessing the accuracy of postoperative implant placement (Contribution 5). Traditional 2D X-ray imaging is readily available; however, there is a paucity of data evaluating whether 2D radiographs can be accurately used to compare postoperative positioning with CT-based preoperative plans. Surprisingly, the results of their study showed that conventional 2D X-rays are adequate for assessing implant placement when using the proposed overlay technique postulated. The introduction of this technique facilitates postoperative follow-up examinations, and in addition reduces the amount of ionizing radiation needed to confirm accurate implant placement.

Taken together, the relevant results from the studies included in this Special Issue highlight the diversity in the field of MSDs and further corroborate how each advancement is a piece in a puzzle that will ultimately help to further advance patient care in the field of MSDs.
